# Tractostorm: The what, why, and how of tractography dissection reproducibility

**DOI:** 10.1002/hbm.24917

**Published:** 2020-01-10

**Authors:** Francois Rheault, Alessandro De Benedictis, Alessandro Daducci, Chiara Maffei, Chantal M. W. Tax, David Romascano, Eduardo Caverzasi, Felix C. Morency, Francesco Corrivetti, Franco Pestilli, Gabriel Girard, Guillaume Theaud, Ilyess Zemmoura, Janice Hau, Kelly Glavin, Kesshi M. Jordan, Kristofer Pomiecko, Maxime Chamberland, Muhamed Barakovic, Nil Goyette, Philippe Poulin, Quentin Chenot, Sandip S. Panesar, Silvio Sarubbo, Laurent Petit, Maxime Descoteaux

**Affiliations:** ^1^ Sherbrooke Connectivity Imaging Laboratory (SCIL) Université de Sherbrooke Sherbrooke Canada; ^2^ Neurosurgery Unit, Department of Neuroscience and Neurorehabilitation Bambino Gesù Children's Hospital, IRCCS Rome Italy; ^3^ Computer Science Department University of Verona Verona Italy; ^4^ Athinoula A. Martinos Center for Biomedical Imaging Massachusetts General Hospital and Harvard Medical School Boston MA; ^5^ Cardiff University Brain Research Imaging Centre (CUBRIC), School of Psychology Cardiff University Cardiff UK; ^6^ Signal Processing Lab (LTS5) École Polytechnique Fédérale de Lausanne Lausanne Switzerland; ^7^ Department of Neurology University of California San Francisco CA; ^8^ Imeka Solutions Sherbrooke Canada; ^9^ Départment de neurochirurgie Hôpital Lariboisière Paris France; ^10^ Department of Psychological and Brain Sciences Indiana University Bloomington IN; ^11^ UMR 1253, iBrain Université de Tours, Inserm Tours France; ^12^ Brain Development Imaging Laboratories, Department of Psychology San Diego State University San Diego CA USA; ^13^ Learning Research & Development Center (LRDC) University of Pittsburgh Pittsburgh PA USA; ^14^ ISAE‐SUPAERO Toulouse France; ^15^ Department of Neurosurgery Stanford University Standford CA; ^16^ Division of Neurosurgery, Emergency Department, "S. Chiara" Hospital Azienda Provinciale per i Servizi Sanitari (APSS) Trento Italy; ^17^ Groupe d'Imagerie Neurofonctionnelle, Institut des Maladies Neurodégénératives ‐ UMR 5293, CNRS CEA University of Bordeaux Bordeaux France

**Keywords:** bundle segmentation, diffusion MRI, inter‐rater, intra‐rater, reproducibility, tractography, white matter

## Abstract

Investigative studies of white matter (WM) brain structures using diffusion MRI (dMRI) tractography frequently require manual WM bundle segmentation, often called “*virtual dissection*.” Human errors and personal decisions make these manual segmentations hard to reproduce, which have not yet been quantified by the dMRI community. It is our opinion that if the field of dMRI tractography wants to be taken seriously as a widespread clinical tool, it is imperative to harmonize WM bundle segmentations and develop protocols aimed to be used in clinical settings. The EADC‐ADNI Harmonized Hippocampal Protocol achieved such standardization through a series of steps that must be reproduced for every WM bundle. This article is an observation of the problematic. A specific bundle segmentation protocol was used in order to provide a real‐life example, but the contribution of this article is to discuss the need for reproducibility and standardized protocol, as for any measurement tool. This study required the participation of 11 experts and 13 nonexperts in neuroanatomy and “*virtual dissection*” across various laboratories and hospitals. Intra‐rater agreement (Dice score) was approximately 0.77, while inter‐rater was approximately 0.65. The protocol provided to participants was not necessarily optimal, but its design mimics, in essence, what will be required in future protocols. Reporting tractometry results such as average fractional anisotropy, volume or streamline count of a particular bundle without a sufficient reproducibility score could make the analysis and interpretations more difficult. Coordinated efforts by the diffusion MRI tractography community are needed to quantify and account for reproducibility of WM bundle extraction protocols in this era of open and collaborative science.

## 1 INTRODUCTION

Diffusion MRI (dMRI) tractography reconstructs streamlines that model the white matter (WM) neuroanatomy. The set of all streamlines forms an object often called the tractogram (Catani & De Schotten, 2008; Jeurissen, Descoteaux, Mori, & Leemans, 2017). When specific hypotheses about known pathways, that is, WM bundles, are investigated, neuroanatomists design “*dissection plans*” that contain anatomical landmarks and instructions to isolate the bundle of interest from this whole brain tractogram (Bayrak et al., 2019; Catani & De Schotten, 2008; Catani, Howard, Pajevic, & Jones, 2002; Chenot et al., 2019; Hau et al., 2016). From now on “*dissection plans*” will be referred as segmentation protocols. Bundles can be segmented to study WM morphology, asymmetries, and then can be associated with specific functions (Catani et al., 2007; Groeschel et al., 2014; Lee Masson, Wallraven, & Petit, 2017; Masson, Kang, Petit, & Wallraven, 2018) with approaches similar to other brain structures (Lister & Barnes, 2009; Reitz et al., 2009). Despite having similar anatomical definitions across publications, the absence of common segmentation protocols for tractography leads to differences that are for the most part unknown and unaccounted for. We need to know how variable our measurements are if we want to be able to have robust bundle‐based statistics in the future. At the moment, there are no standardized method being used by the community.

The need for a gold standard that quantifies human variability is well‐known and well‐studied in other fields, such as automatic image segmentation, cell counting, or in machine learning (Boccardi et al., 2011; Entis, Doerga, Barrett, & Dickerson, 2012; Kleesiek et al., 2016; Piccinini, Tesei, Paganelli, Zoli, & Bevilacqua, 2014). For applications such as hippocampi or tumor segmentation, thorough assessments of reproducibility and multiple iterations of manual segmentation protocols already exist (Boccardi et al., 2015; Frisoni et al., 2015). These protocols were specifically designed to reduce the impact of human variability and help outcome comparison in large‐scale clinical trials across multiple centers (Frisoni et al., 2015; Gwet, 2012). It is our opinion that the very same steps are needed for every WM pathways in order to achieve reproducible results. Our work is, in fact, an attempt to expose and clarify the necessity to design harmonized protocols, quantify their reproducibility and take variability into account when reporting results.

The reproducibility of manual bundle segmentation is likely to be always lower than manual image segmentation. Image segmentation in 3D requires local decision‐making, and the decision to include voxels or not is directly done by raters. However, bundle segmentation requires local decisions that possibly impact the whole volume as streamlines reach outside of the scope of decisions made by raters. Since small or large hand‐drawn regions of interest (ROIs) or spheres are used to segment bundles, small mistakes can have far‐reaching consequences. Even if ROIs are fairly reproducible in a strict protocol, the resulting bundles could be far from reproducible. This local‐decision and global‐impact conundrum makes the design of reproducible protocols more difficult and can potentially cause low agreement between raters.

### 1.1 Bundle segmentation

**Figure 1:**
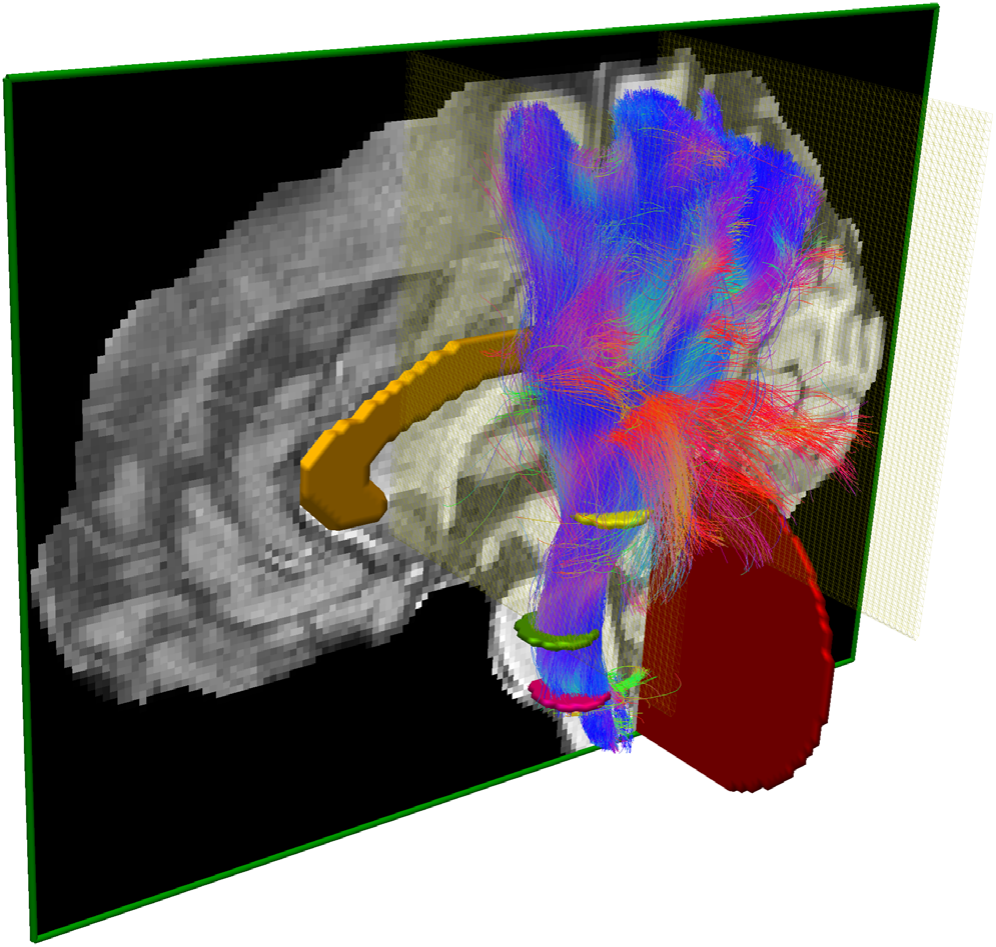
Illustration of the dissection plan of the PyT using the MI‐Brain software (Rheault, Houde, Goyette, Morency, & Descoteaux, 2016). Three axial inclusion ROIs (pink, green, yellow), one sagittal exclusion ROIs (orange), two coronal exclusion ROIs (light yellow), and a cerebellum exclusion ROIs (red, optional). The whole brain tractogram was segmented to obtain the left PyT. PyT, pyramidal tract; ROIs, regions of interest

Bundle segmentation is the action of isolating streamlines based on neuroanatomical priors, using known regions where certain conditions need to be satisfied. Inclusion and exclusion ROIs are drawn and defined at the voxel‐level using coregistered structural images and are subsequently used to select the streamlines produced by tractography (Behrens, Berg, Jbabdi, Rushworth, & Woolrich, 2007; Catani et al., 2002; Ghaziri et al., 2015; Renauld, Descoteaux, Bernier, Garyfallidis, & Whittingstall, 2016; Rozanski et al., 2017), as shown in Figure 1. Streamlines can be included or discarded using inclusion ROIs where streamlines are forced to traverse, and exclusion ROIs that cannot be crossed. Known structures such as gray nuclei, gyri, or sulci and recognizable signal signatures can be used as landmarks to create a plan to follow for the segmentation (Catani et al., 2002; Catani & De Schotten, 2008; Chenot et al., 2019; Hau et al., 2016). In this work, the person performing the task of segmentation (i.e., drawing the ROIs, following the protocol) will be referred to as *rater*. Manual segmentation can be performed in software such as, but not limited to, DTI studio (Jiang, Van Zijl, Kim, Pearlson, & Mori, 2006), Trackvis (Wang, Benner, Sorensen, & Wedeen, 2007), exploreDTI (Leemans, Jeurissen, Sijbers, & Jones, 2009), MITK Diffusion (Neher et al., 2012), FiberNavigator (Chamberland, Whittingstall, Fortin, Mathieu, & Descoteaux, 2014), or MI‐Brain (Rheault et al., 2016) (Figure 1).

Once a bundle of interest is segmented from a tractogram, the analysis varies according to the research question. It is common to report asymmetry or group difference in bundle volume (Catani et al., 2007; Chenot et al., 2019; Song et al., 2014), diffusion values within the bundle of interest (average fractional anisotropy, mean diffusivity, etc.) (De Erausquin & Alba‐Ferrara, 2013; Kimura‐Ohba et al., 2016; Ling et al., 2012; Mole et al., 2016) or values along the bundle (called *profilometry* and *tractometry*) (Cousineau et al., 2017; Dayan et al., 2016; Yeatman, Dougherty, Myall, Wandell, & Feldman, 2012; Yeatman, Richie‐Halford, Smith, Keshavan, & Rokem, 2018). Spatial distribution of cortical terminations of streamlines can help to identify cortical regions with underlying WM connections affected by a condition (Behrens et al., 2003; Donahue et al., 2016; Johansen‐Berg et al., 2004; Mars et al., 2011; Rushworth, Behrens, & Johansen‐Berg, 2005). Reporting the number of streamlines (e.g., streamline count in connectivity matrix or density maps) is still very much present as a way to compare groups (Girard, Whittingstall, Deriche, & Descoteaux, 2014; Jones, Knösche, & Turner, 2013; Sotiropoulos & Zalesky, 2017), despite not being directly related to anatomy or connection strength (Jones, 2010; Jones et al., 2013).

### 1.2 Quantifying reproducibility in tractography

When performing segmentation, it is crucial that raters perform the tasks as closely as possible to the dissection plan. Even if a single individual performs all segmentations, the possibility of mistakes or erroneous decisions about landmarks exists (Boccardi et al., 2011; Entis et al., 2012; Frisoni et al., 2015). High reproducibility is often an assumption, if this assumption is false, the consequence could lead to inconsistent outcomes and erroneous conclusions. To assess the level of reproducibility of raters, identical datasets need to be segmented blindly more than once (Frisoni et al., 2015; Gisev, Bell, & Chen, 2013; Gwet, 2012). The literature on the subject, specifically for tractography, is quite sparse. Reported values for intra‐rater and inter‐rater variability are sometimes hidden in Section 2. However, it is common to report measures such as volume or average FA, which do not directly relate to spatial agreement (as detailed in Section 2.4), or to report variability of the ROI drawn by raters instead of the resulting bundles. Finally, the steps to perform the segmentation (the protocol) are not provided and the framework for evaluation is not defined. Despite these limitations, the general trend is that different bundles do not have the same level of variability (ranging from 0.4 to 0.95) and that algorithmic choices (e.g., diffusion tensor imaging vs. high angular resolution diffusion imaging) have an influence on variability (Colon‐Perez et al., 2016; Dayan, Kreutzer, & Clark, 2015; Kaur, Powell, He, Pierson, & Parikh, 2014; Kreilkamp et al., 2019; Voineskos et al., 2009; Wakana et al., 2007; Yendiki et al., 2011). The first, and probably most complete, publication on the subject of protocol reproducibility assessment was from Wakana et al. (2007). The acquisition and algorithmic choices for tractography could be considered suboptimal (low spatial/angular resolution, diffusion tensor) and the framework inadequate for the now more common large‐scale collaborations. The publication, despite providing a robust design, had a limited number of raters and duplicated data. The importance of reproducibility assessment, sparse literature on the subject, and limited availability of protocols support the need for the work presented in this study.

To come back to tractography, the main message of our work is simple: Any study involving a manual segmentation protocol must provide a quantification of its measurement error, if it was never assessed before. Any modifications to the experimental setup will require a new assessment of the measurement error. Reporting measurements, such as average fractional anisotropy (FA), volume, or streamline count, without a sufficient reproducibility is potentially problematic. Too low of an agreement score (e.g., below 30%) could even mean an entirely different BOI/ROI is segmented, which would hinder further analysis. Similarly to the The EADC‐ADNI Harmonized Hippocampal Protocol (HarP) (Frisoni et al., 2015), future dMRI tractography protocols will have to be designed for each bundle of interest. Groups of experts will have to propose protocols, pick‐and‐choose the best features of each and design an agreed upon set of rules, as it was undertaken for the HarP project (Boccardi et al., 2015). As of this moment, efforts are being made toward an inter‐protocol variability study to evaluate the current state of anatomical definitions present in the field, similar to the HarP project (Boccardi et al., 2011). In this work, measurement error is sometimes referred as variability, while reproducibility is the capacity to reach the same results twice. Reproducibility of segmentations from the same individual is referred to as intra‐rater agreement, while reproducibility of segmentation across raters is referred to as inter‐rater agreement.

**Figure 2:**
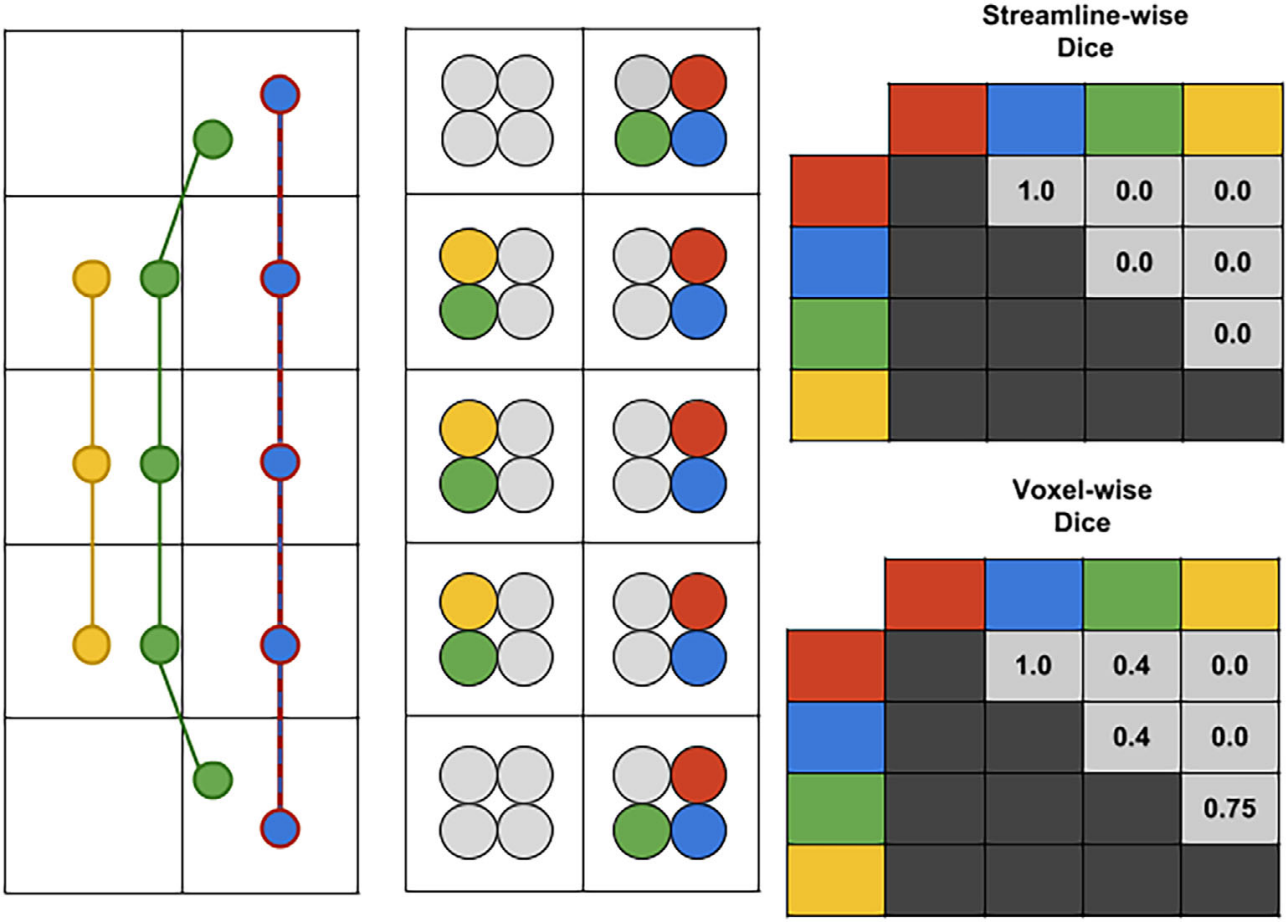
Representation of the Dice Coefficient (overlap) for both the streamline and the voxel representation. For the purpose of a didactic illustration, four streamlines are showed in a 2×5 “voxel grid,” the red and blue streamlines are identical. Each streamline is converted to a binary mask (point‐based for simplicity) shown in a compact representation. Voxels with points from three different streamlines will results in voxels with three different colors, this can be seen as a spatial smoothing. The matrices on the right show values for all pairs (symmetrical). The green and yellow streamline are not identical, which results in a streamline‐wise Dice coefficient of zero. However, in the voxel representation they have three voxels in common and the result is $\left(\frac{2\times 3}{5+3}=0.75\right)$

In the field of neuroimaging, voxels are used as the typical representation of data, while the available representation in tractography is in the form of streamlines (i.e., sets of 3D points in space). Figure 2 is a sketch of both representation. Several similarity measures exist to compare voxel‐wise segmentations, for example, Dice score. Most of them have an equivalent formulation to compare sets of streamlines. However, resulting values can widely vary as the spatial distribution is not the same for both representations. Some measures related to streamlines require the datasets to be exactly the same, for example, Dice score, as streamline reconstructions are sets of discrete points with floating point coordinates and not discrete grids like 3D images. For this reason, comparison of streamlines is more challenging and datasets that do not originate from the same source distance in millimeters is often the only available solution (Garyfallidis et al., 2017; Maier‐Hein et al., 2017). Automatic segmentation methods are becoming more widespread. Methods such as, but not limited to, (Chekir, Descoteaux, Garyfallidis, Côté, & Boumghar, 2014; Garyfallidis et al., 2017; Guevara et al., 2011; O'Donnell et al., 2017; O'donnell, Golby, & Westin, 2013; Wassermann et al., 2016; Wasserthal, Neher, & Maier‐Hein, 2018; Yendiki et al., 2011; Zhang et al., 2018) aim to simplify the work of raters. The typical standard of most automatic segmentation method is to reach the accuracy of raters, thus it is crucial to truly quantify human reproducibility in manual tasks. It is possible to envision a scenario where an automatic method would not be as accurate as human expertise but still useful to provide insight or even valid biomarkers. In such a case, it is still useful to know how accurate human expertise is, at least to provide comparisons.

### 1.3 Summary of contributions of this work

Our PyT evaluation experiment serves as an “*example*” to convey the point that tractography segmentation is not standardized and needs to be addressed properly to make it quantitative, robust, and more useful in the neuroimaging and human brain mapping literature.

We propose a framework to evaluate and quantify human reproducibility of bundle segmentation from dMRI tractography. Obtaining a measurement of rater (intra and inter) agreement is extremely relevant to set an appropriate threshold for statistical significance. It is also relevant for meta‐analysis aiming to study large sets of publications and synthesize their outcomes. An account of human errors or other sources of variability is necessary. The main goal of this publication is to promote the importance of the assessment of any new “*virtual dissection*” protocol. We do not want to promote a given dissection protocol but emphasize the fact that any new protocol, including a new tractography algorithm and another bundle of interest would require a new assessment.

A second contribution of this work is to investigate overlap, similarity measures, and gold standard comparison designed for tractography. Development of easily interpretable measures for bundle comparison is necessary for large datasets. Overall, the voxel representation is significantly more reproducible than the streamline representation. The voxel representation is better suited for analysis of tractography datasets (e.g., reporting volume instead of streamline count). More details about these different representations and voxel/streamline‐wise measures will be detailed in Sections 2 and 3.

## 2 METHOD

### 2.1 Study design

Twenty‐four participants were recruited and divided into two groups: experts and nonexperts. The division was based on their neuroanatomical educational background. Participants working as researchers or PhD students in neuroanatomy, neurology or with extended experience in the field performing “*virtual dissection*” as well as neurosurgeons were part of the experts group (11 participants). The nonexperts group was composed of MSc, PhD student or PostDoc in neuroimaging, but without any formal education in neuroanatomy (13 participants). All participants had knowledge of dMRI tractography in general as well as the concept of manual segmentations of tractography datasets. Participation was voluntary and anonymous, recruitment was done individually and participants from various laboratories in Europe and the United States were solicited. The study was performed according to the guidelines of the Internal Review Board of the Centre Hospitalier Universitaire de Sherbrooke (CHUS).

Five independent tractograms and their associated structural/diffusion images were used, each was triplicated (total of 15). One was untouched, one was flipped in the X‐axis (left/right), and one was translated. This was done to ensure that the participants were not aware they were performing reproducibility tasks. The symmetry of the segmentation plan (no difference between hemispheres) and lack of absolute frame of reference (coordinates) allowed these operations. Then, all datasets were randomly named so the tasks could be performed blindly for each participant. Participants were not aware of the presence of duplicated datasets. Five tractotrams were used to obtain stable averages, duplicated datasets were used to score the intra‐rater agreement and the multiple participants to evaluate inter‐rater agreement. The decision to separate participants in two groups was made to generate additional data about reproducibility in real‐life conditions.

**Figure 3:**
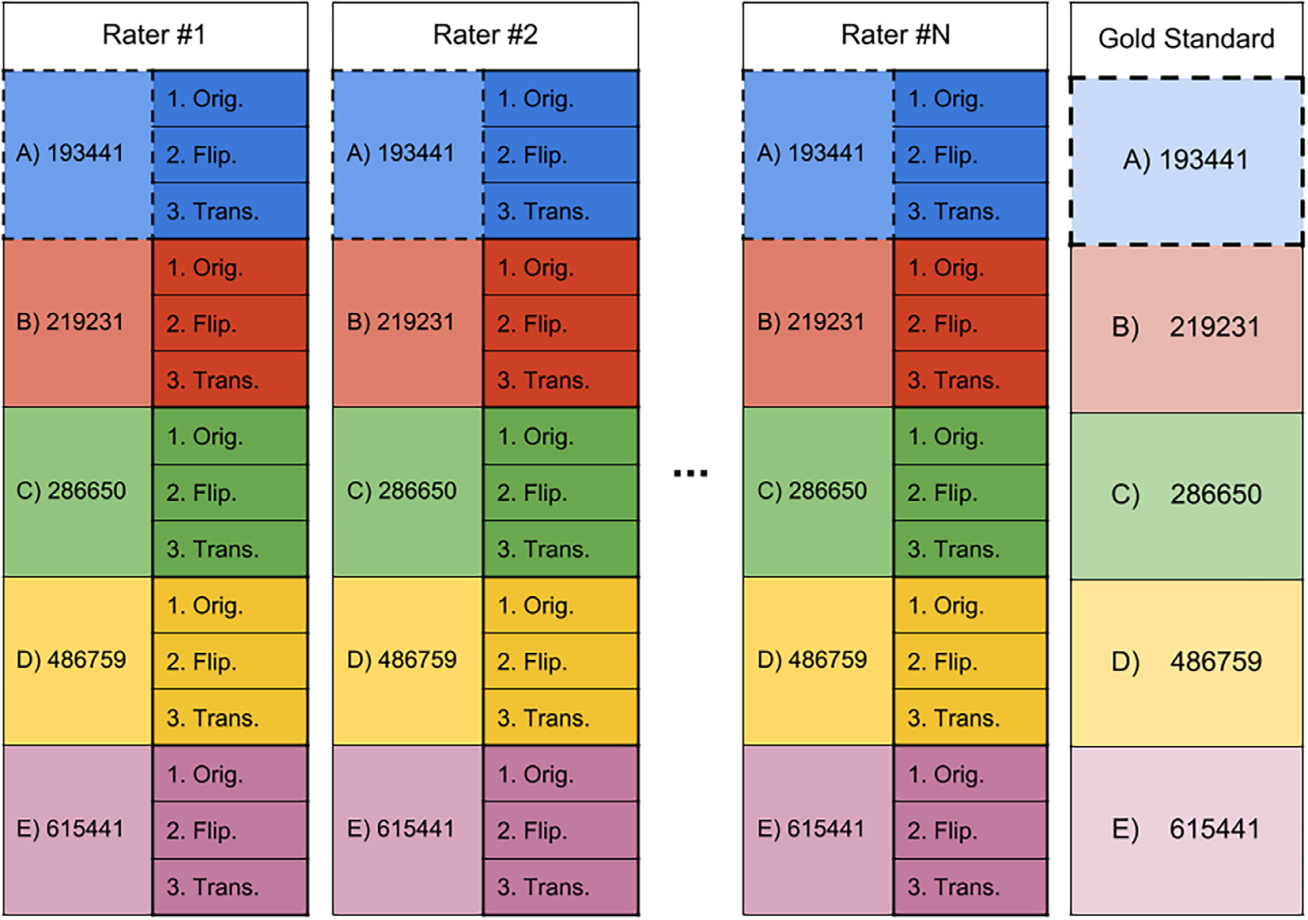
Representation of the study design showing *N* participants, each received five HCP datasets (listed and color coded) which were replicated three times (original, flipped, translated). All participants had to perform the same dissection tasks, on the same anonymized datasets. Intra‐rater, inter‐rater, and gold standard reproducibility were computed using the deanonymized datasets. More details are available in the Supporting Information

Figure 3 shows an overview of the study design. To evaluate intra‐rater reproducibility of rater #1, each triplicate was used to compute reproducibility measures. Meaning that 5 (A‐B‐C‐D‐E) × 3 (1–2‐3) values were averaged to obtain the intra‐rater “*reproducibility score*” of a single rater. To evaluate inter‐rater reproducibility of rater #1, triplicates were fused and compared to all other raters to obtain a reproducibility measure. Meaning that 5 (A‐B‐C‐D‐E) × N (raters) values were averaged to obtain a single inter‐rater “*reproducibility score*.” To evaluate the reproducibility of rater #1 against the gold standard, the fused triplicates were also used. Meaning that 5 (A‐B‐C‐D‐E) × 1 (gold standard) values were averaged to obtain a single rater gold standard “*reproducibility score*.” The results shown in Section 3 are average values from all raters in each group. All reproducibility measures were computed using the same approach.

### 2.2 DWI datasets, processing, and tractography

Tractograms were generated from the preprocessed Human Connectome Project (HCP) (Van Essen et al., 2013) diffusion weighted image (DWI) data (three males and two females, healthy, 26–35 years old) using three shells (1,000, 2,000, and 3,000) with 270 directions. The b0, FA and RGB (colored FA) images were computed from DWI to be used as anatomical reference during segmentation. Constrained spherical deconvolution (CSD) using a FA threshold from a tensor fit on the b = 1000 s/mm^2^ was used to obtain fiber orientation distribution functions (fODF) (Descoteaux, Angelino, Fitzgibbons, & Deriche, 2007; Tournier, Calamante, & Connelly, 2007) (spherical harmonic order 8) from the b = 2000 s/mm^2^ and b = 3,000 s/mm^2^ shells. Probabilistic particle filtering tractography (Girard et al., 2014) was subsequently computed at 30 seeds per voxel in the WM mask (FSL FAST [Woolrich et al., 2009]) to make sure sufficient density and spatial coverage were achieved. The decision to use high‐quality data was to maximize the quality of the tractogram so it would not be a limiting factor for the segmentation tasks. Moreover, it is now more and more common in clinical research to reach resolution as high as 1.5 mm isotropic with multishell schemes, such dataset generates tractograms on a similar quality range as the HCP.

The CSD model was also used for bundle‐specific tractography (BST) to further improve density and spatial coverage of the bundle of interest (Rheault et al., 2019). This was to ensure that the full extent of the PyT was reconstructed and to ensure not to have criticisms from our experts in neuroanatomy complaining of a lack of fanning (Pujol et al., 2015). A large model that approximates the corticospinal tracts (CST), which encompass the PyT, was used to generate streamlines with a strong preference for the Z‐axis (up‐down). A similar PyT reconstruction could have been achieved by generating millions and millions of streamlines, which would have been heavy and cumbersome from dissectionnists participants in the study. This approach was used to increase the PyT reconstruction quality in (Chenot et al., 2019) and demonstrated its usefulness.

Furthermore, only the general orientational priors was used (globally helping the *up/down* orientation) during tractography and not spatial/tissue priors was used. Meaning that globally results in a whole brain tractogram that was seeded from tens of thousands of voxels from a large region apparent to the CST. And these results were fused to a conventional whole brain CSD probabilistic tractogram. The resulting tractogram provided to the participants is indistinguishable from a conventional one. The rationale for this decision to use a more efficient seeding/tracking method to fill‐up the full spatial extent of the PyT, as opposed to generating 10–50 millions of streamlines.

To accommodate all participants and the wide range of computer performance, tractograms were compressed using a 0.2 mm tolerance error (Presseau, Jodoin, Houde, & Descoteaux, 2015; Rheault, Houde, & Descoteaux, 2017) and commissural streamlines were removed and datasets split into hemispheres. Each hemisphere (of each subject) had approximately 500,000 streamlines.

### 2.3 Dissection plan and instructions

Each participant received their randomly named datasets, a document containing instructions for the segmentation and a general overview of a segmentation as an example (see Supplementary Information). The segmentation task consisted in 15 segmentations of the pyramidal tract (left and right). The rationale behind the decision to focus on this PyT bundle was first, that a well‐defined and well‐known pathway was desired. Second, a dissection plan made of small and large inclusion and exclusion regions was desired. Finally, that the general shape was intuitive so the participants with no background in neuroanatomy could perform the tasks. Segmentation involved using three WM inclusion ROIs (internal capsule, midbrain, and medulla oblongata) and two exclusion ROIs (one plane anterior to the precentral gyrus and one plane posterior to the postcentral gyrus). The detailed segmentation plan is available in the Supporting Information (Chenot et al., 2019).

Participants had to perform the segmentation plans, following the instructions as closely as possible. The dataset order was provided in a spreadsheet file. Participants had to choose between two software; Trackvis (Wang et al., 2007) or MI‐Brain (Rheault et al., 2016). This decision was made to guarantee that the data received from all participants was compatible with the analysis. Metadata such as date, starting time, and duration had to be noted in the spreadsheet file. Upon completion, the participants had to send back the same 15 folders with two tractography files in each, the left and right pyramidal tract (PyT).

### 2.4 Bundles analysis

Once returned by all participants, datasets were de‐randomized to match triplicates across participants. The duplicates (flipped and translated) were reverted back to their native space and all datasets (images and tractograms) were warped to a common space (MNI152c 2009 nonlinear symmetrical) using the Ants registration library (Avants, Epstein, Grossman, & Gee, 2008; Fonov et al., 2011) to simplify the analysis. With all datasets having a uniform naming convention and in a common space, the intra‐rater and inter‐rater reproducibility can be assessed.

#### 2.4.1 Individual measures

Reproducibility can be assessed using various measures. Average FA, volume, and streamline count are the main attributes obtained directly from files. They do not provide direct insight about reproducibility, but one could expect that very similar segmentations should have very similar values. However, segmentation could wildly differ across rater and yet these measurements could be very similar. Average FA, volume, or streamline count comparison do not provide any information about reproducibility. Reporting values from bundles obtained via manual segmentation using a protocol with unknown reproducibility scores is uninterpretable. This is simply due to the fact that completely different bundles can have the same measurements. This is why a confirmation that raters following the same protocol obtain the “*same*” segmentation is crucial, that is, high reproducibility. In this work, results for the left and right PyT are averaged together without distinction, they are considered the same bundle during the analysis.

#### 2.4.2 Intra‐rater and inter‐rater

**Figure 4:**
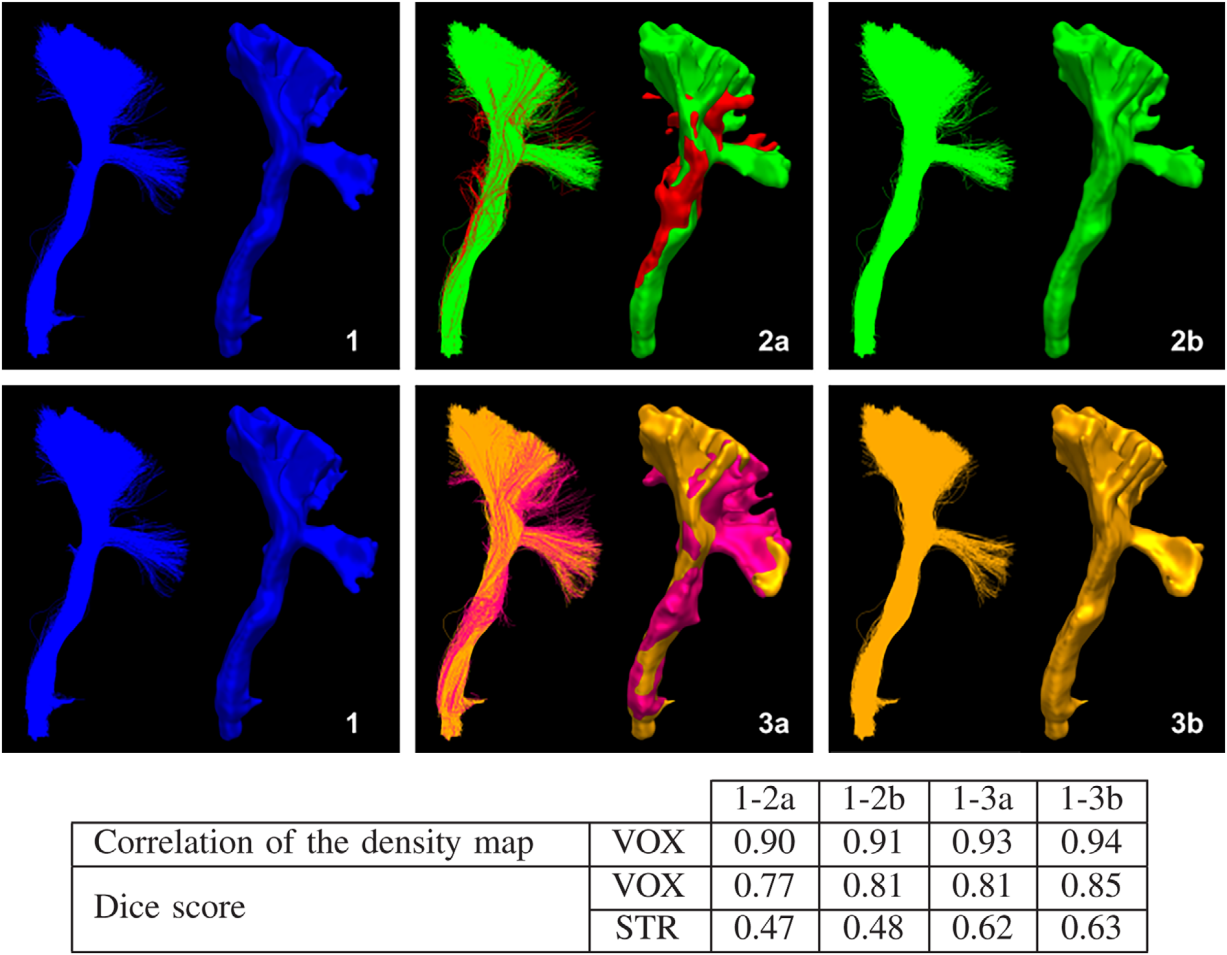
Comparison of bundles and the impacts of spurious streamlines on the reproducibility measurements. Each block shows streamlines on the left and the voxel representation on the right (isosurface). Block 2a and 3a shows the core (green/orange) and spurious (red/pink) portion of the bundle. Block 2b and 3b only shows the core portion of the bundle. Table showing the reproducibility “score” between bundles, VOX marks voxel‐wise measures, and STR marks streamlinewise measures

Each participant performed the same tasks on each triplicate. The goal of this triplication is to evaluate intra‐rater reproducibility. Since all participants had access to the same datasets, inter‐rater reproducibility can be assessed too. Figure 4 shows the effect of of spurious streamlines in segmentation on reproducibility measurements.

Computing the average value from all pairwise combinations provides an estimate of the agreement between multiple segmentations of a same bundle. The deviation can also provide insights about the consistency of these segmentations. Measurement values can be between 0 and 1, such as Dice and Jaccard (Dice, 1945), meaning they are independent of the size. Figure 4 shows bundles and how to interpret these measures. Pearson's correlation coefficient obtained from density maps provides insight into the statistical relationship and spatial agreement between two segmentations (Hyde & Jesmanowicz, 2012). Each measure provides a way to interpret the data at hand, but there is no single true measure to summarize intra‐rater and inter‐rater agreement.

#### 2.4.3 Gold standard

When multiple raters provide segmentations from an identical dataset, it is of interest to produce a gold standard. For a voxel representation, a probability map can be constructed, where each voxel value represents the number of raters that counted the voxel as part of their segmentation (Frisoni et al., 2015; Iglesias & Sabuncu, 2015; Langerak, van der Heide, Kotte, Berendsen, & Pluim, 2015; Pipitone et al., 2014). This can be normalized and then thresholded to obtain a binary mask representing whether or not the voxel was segmented by enough rater. A threshold above 0.5 is often referred to as a majority vote. The same logic can be applied to streamlines, each streamline can be assigned a value based on the number of raters that considered it part of their segmentation.

**Figure 5:**
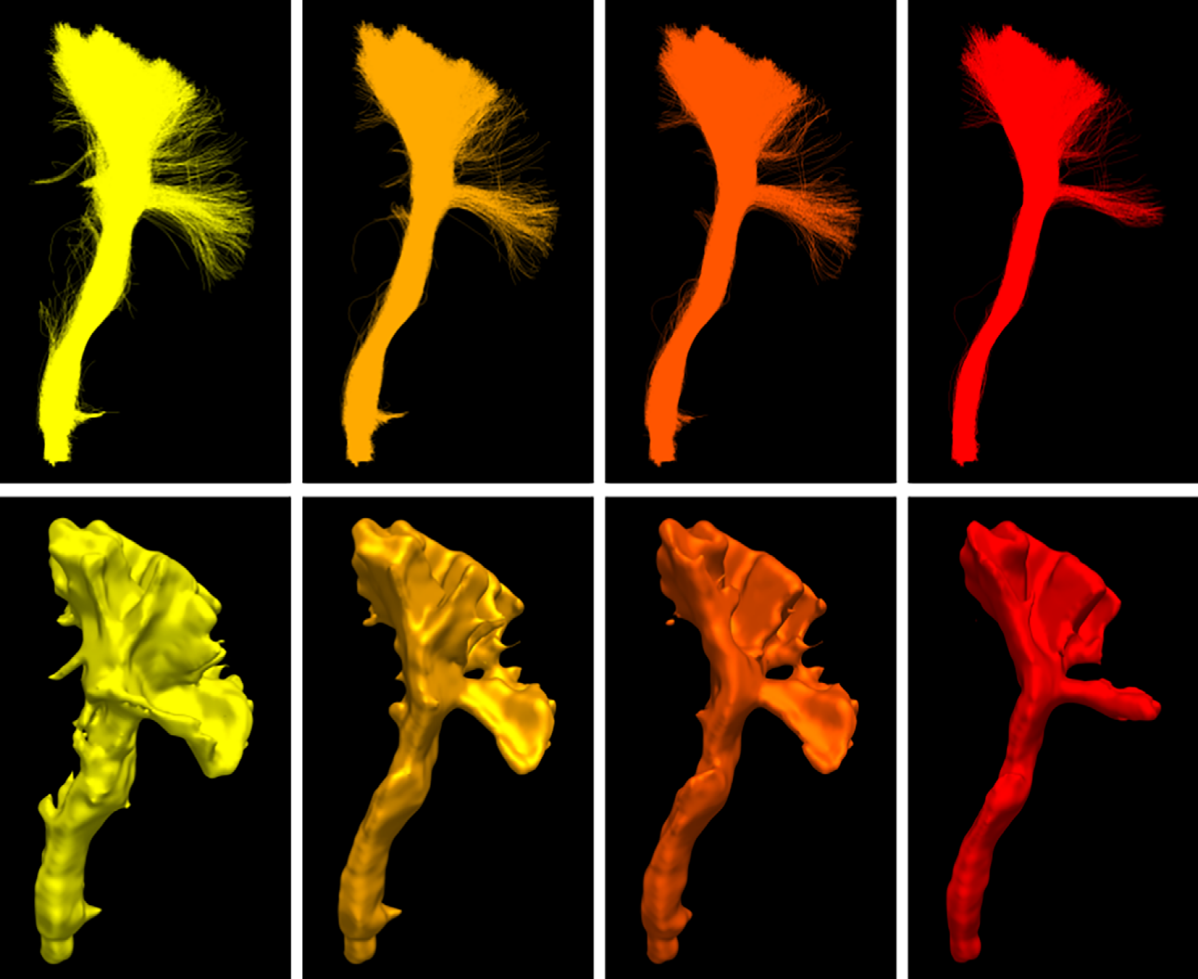
Example of average segmentation, or gold standard, generation obtained from seven different segmentations, first row shows the streamline representation and the second row shows the voxel represented as a smooth isosurface. From left to right, multiple voting ratios were used $\left(\frac{1}{7},\frac{3}{7},\frac{5}{7},\frac{7}{7}\right)$, each time reducing the number of streamlines and voxels consider part of the average segmentation. A minimal vote set at one out of seven (left) is equivalent to a union of all segmentations while a vote set at seven out of seven (right) is equivalent to an intersection between all segmentations

This can be seen in Figure 5 where increasing the minimal vote threshold reduces the number of outliers and overall size. In this work, the gold standard *does not* represent the true anatomy and should not be interpreted as such. It simply represents the average segmentation obtained from a tractogram. All elements that are not in a gold standard are true negatives and all the ones present are true positives. By construction, the gold standard does not contain false positives or false negatives. Binary classification measures are available such as sensitivity or specificity. However, several other measures are available and each is a piece of the puzzle leading to a more accurate interpretation (Chang, Zhuang, Valentino, & Chu, 2009; Garyfallidis et al., 2017; Schilling et al., 2018).

To produce our gold standard, a majority vote approach was used from the segmentations of the experts group, as their knowledge of anatomy was needed to represent an average version of the bundle of interest. In the context of this work, the *gold standard* is actually the average segmentation from experts. For simplicity, the expression *gold standard* was used as it is the best approximation of what can/could be achieved by our group of experts. The vote was set at 6 out of 11 and each of the five datasets got its own left and right gold standard. Since the representation at hand is streamlines (which can be converted to voxels), a streamline‐wise and a voxel‐wise gold standard was created. A majority vote approach is not necessarily optimal, but in the context where experts could not collaborate beforehand or after, this approach is adequate to obtain an average representation of the segmentation.

## 3 RESULTS

In the following sections, all reported values are medians and interquartile ranges (IQR). This choice was made based on the fact that distributions are often bounded and not Gaussian distributions. Captions of figures report results as (*Q*_2_ [*median*]; *Q*_3_ − *Q*_1_ [*IQR*]), with a star (*) indicating if the distributions are significantly different. All explicit comparisons between groups are statistically significant using a Mann–Whitney rank nonparametric test for two independent samples (*p* < .01).

**Figure 6:**
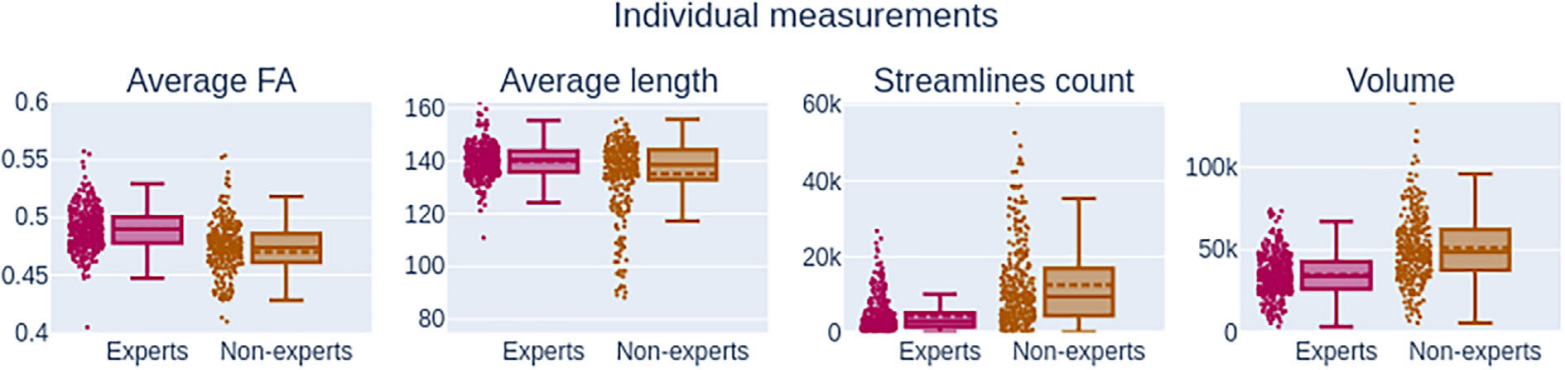
Measurements (*Q*_2_; *IQR*) related to individual files for both groups. The Average FA distribution for experts (0.49; 0.01) and nonexperts (0.47;0.03) is not statistically different from each other. Similarly, the average length of experts (140.33 mm; 7.81 mm) and nonexperts (138.70 mm; 11.29 mm) cannot be distinguished. Streamlines count of experts (2,893; 3564*) has a significant difference of distribution from nonexperts (9,383; 12,368*). The same can be same from the volume distribution (34.00 cm^3^; 16.43 cm^3^*) for experts and (48.74 cm^3^; 24.57 cm^3^*) for nonexperts. The lower and higher fences for nonexperts are much wider, indicating more variation in results

**Figure 7:**
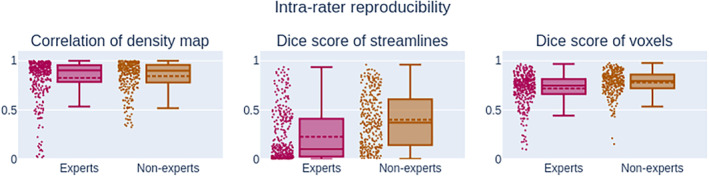
Measurements (*Q*_2_; *IQR*) related to pairwise comparison measures for intra‐rater segmentations. The correlation of density maps showed no statistically significant difference between the experts (0.90; 0.17) and the nonexperts (0.90; 0.17) groups. Distributions showed statistically significant difference for both Dice score. The Dice score of streamlines shows a easily observable difference between experts (0.10; 0.39*) and nonexperts (0.37; 0.46*). The difference between distribution Dice score of voxels is less noticeable at (0.75; 0.15*) for experts and (0.79; 0.14*) for nonexperts. The trend for the intra‐rater reproducibility is that rater fails to select the same streamlines, but the ones that are selected still cover approximately the same volume. IQR: interquartile range

**Figure 8:**
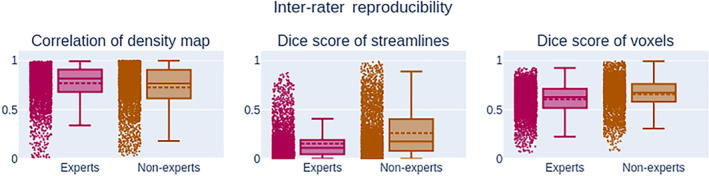
Measurements (*Q*_2_; *IQR*) (*Q*_2_; *IQR*) related to pairwise comparison measures for inter‐rater segmentations. The correlation of density maps showed no statistically significant difference between the experts (0.82; 0.23*) and the nonexperts (0.77; 0.29*) groups. Similarly to the intra‐rater segmentation, distributions showed statistically significant difference for both Dice score. The Dice score of streamlines shows a easily observable difference between experts (0.11; 0.14*) and nonexperts (0.18; 0.32*). While the distribution Dice score of voxels for experts (0.63; 0.20*) and nonexperts (0.67; 0.18*) is more similar. Raters have difficulty to select the same streamlines, but overall capture similar volume. IQR: interquartile range

On average, experts produce “*smaller*” bundles than nonexperts, their volume, and streamline count being lower than nonexperts (−30% and − 60%), as it can be observed in Figure 6. This difference between groups is statistically significant (*p* < .01). The range of values for segmentation measures is wider for nonexperts, meaning that either intra‐rater or inter‐rater variability is likely higher. As mentioned earlier, this is useful insight about reproducibility but lacks nuance and context. For example, despite obvious variation in volume and somewhat poor spatial overlap in segmentations (as shown in Figures 7 and 8), the average FA measurement does not show large variation. The fact that segmentations with low spatial overlap have the same average FA shows that reporting this measurement to gain insight about reproducibility of bundle segmentation is far from optimal. Since bundles without any overlap could have the same average FA, this measure is very difficult to interpret in terms of reproducibility evaluation.

### 3.1 Intra‐rater evaluation

All reported values can be seen in Figure 7. The median intra‐rater overlap is represented by the voxel‐wise Dice coefficient and is 0.75 for experts and 0.79 for nonexperts. Streamline‐wise Dice coefficient is much lower at 0.10 and 0.37 for both groups, respectively. A higher Dice score value means that participants of a group are, on average, more reproducible with themselves. The median density correlation is equal (*p* < .01) at 0.900 for the experts and nonexperts group.

### 3.2 Inter‐rater evaluation

To minimize the influence of intra‐rater reproducibility during the evaluation of inter‐rater reproducibility, the triplicate datasets were fused into a single bundle. This was performed to approximate the results as if participant segmentations had no intra‐rater variability. This leads to an underestimation of inter‐rater variability but necessary to separate sources of variability later in the analysis. Voxel‐wise Dice coefficient is on average higher between experts than between nonexperts, at 0.62 and 0.67, respectively, while the streamline‐wise Dice coefficient is much lower at 0.11 and 0.18. The median density correlation is higher between experts at 0.88 while nonexperts are at 0.71. The IQR is higher for the nonexperts group, meaning that the similarity among nonexperts is not only lower but widely varies. All reported values can be seen in Figure 8.

### 3.3 Gold standard evaluation

**Figure 9:**
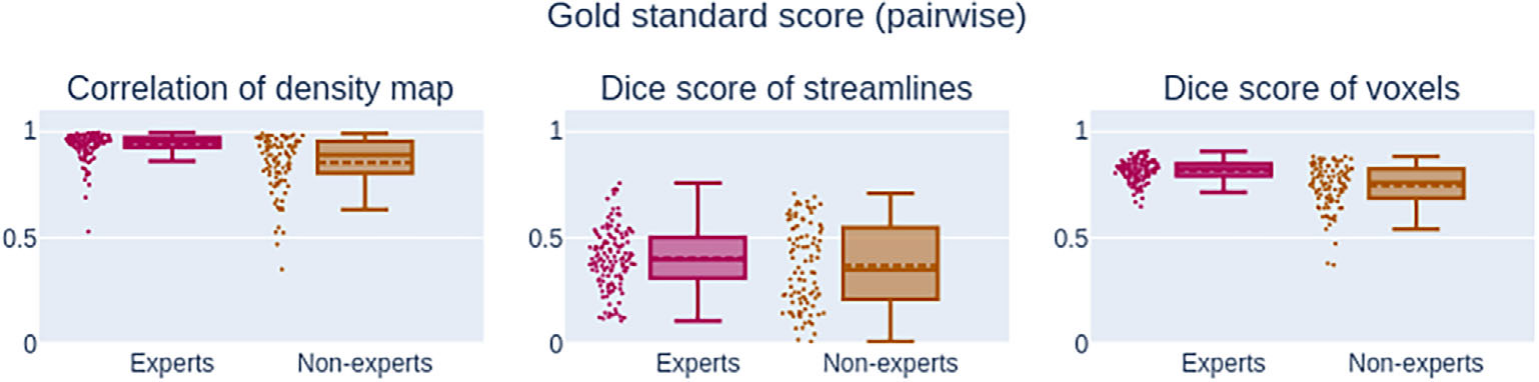
Measurements (*Q*_2_; *IQR*) related to pairwise comparison measures against the gold standard. The correlation of density map reaching (0.95; 0.04*) for experts and (0.88;1 5*) is statistically different between both groups. However, the Dice score of streamlines are not statistically different at (0.39; 0.18) and (0.34; 0.34), respectively. The Dice score of voxel is relatively high at (0.82; 0.05*) for experts and (0.76; 0.13*) for nonexperts. Despite variations between rater, overall the participants remain around the same average segmentation and obtain more agreement with the gold standard than with each other. IQR: interquartile range

**Figure 10:**
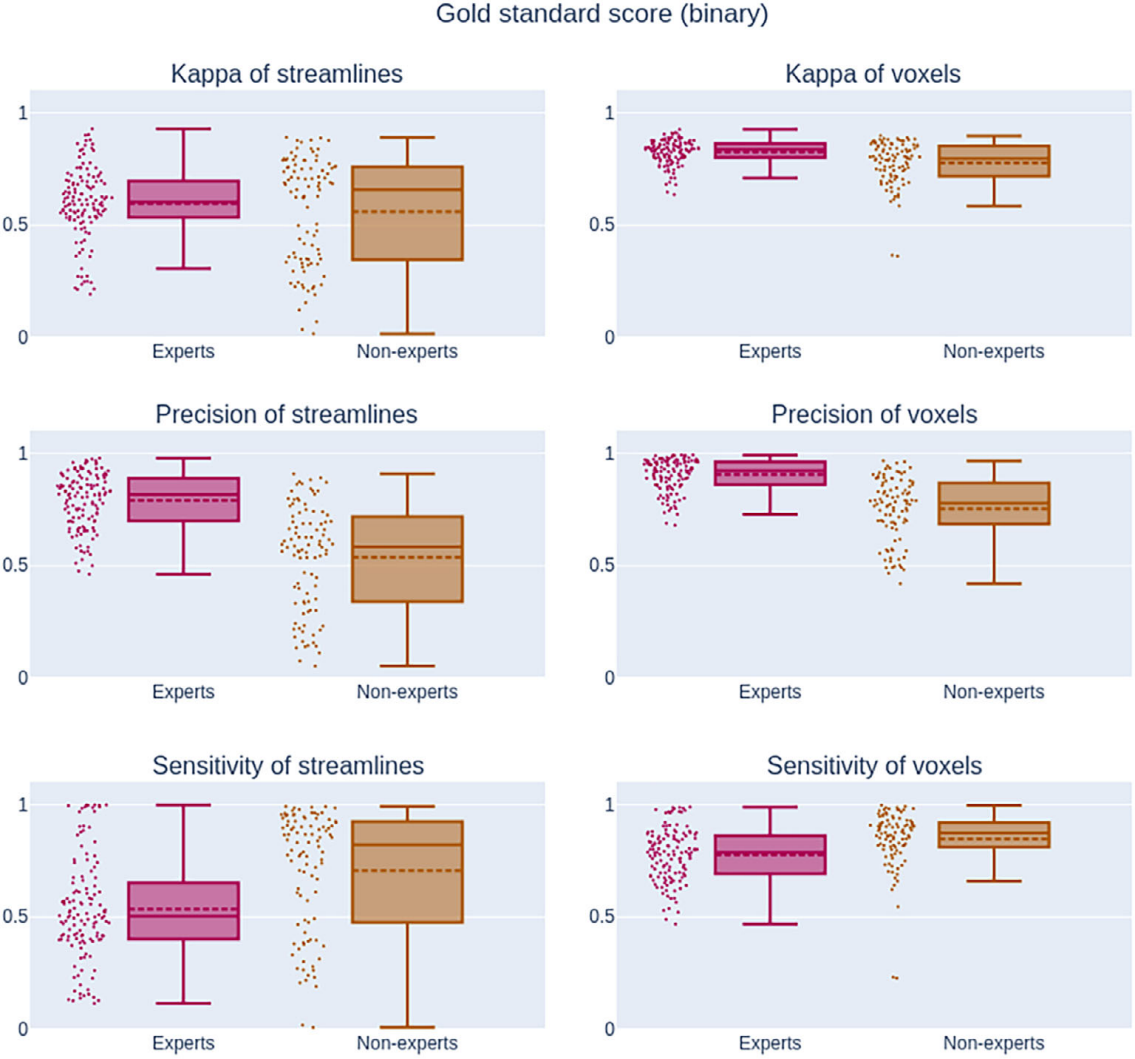
Measurements (*Q*_2_; *IQR*) related to binary classification measures against the gold standard. The Kappa score is only significantly different for voxel (0.84; 0.06 and 0.80; 0.13) and not for streamlines (0.60; 0.16* and 0.65; 0.41*). There is a high degree of variability for precision and sensitivity of streamlines (0.81; 0.19* and 0.50; 0.24* for experts) and (0.59; 0.37* and 0.82; 0.44* for nonexperts). These measures are more reliable with the voxel representation (0.92; 0.10* and 0.79; 0.17* for experts) and (0.78; 0.17* and 0.82; 0.44* for nonexperts). The streamline representation is always less reproducible than the voxel representation. The measures such as accuracy and specificity are not shown due to the fact that both reach above 0.99 and do not provide useful visual insight. IQR: interquartile range

All reported values can be seen in Figures 9 and 10. Comparisons to the computed gold standard show that on average experts and nonexperts obtain segmentation roughly similar to the average segmentation. However, all measures show that segmentations from experts are on average closer to the gold standard than those of nonexperts. This was expected as the gold standard was produced using segmentations from the experts group. Values for streamline‐wise measures are lower for Dice coefficient and density correlation, meaning that reproducibility is harder to achieve using the streamline representation. This was a similar trend observed in intra‐rater and inter‐rater values.

Specificity and accuracy reach above the 95% for both groups both for streamlines or voxels. Meaning that experts and nonexperts alike classified the vast majority of true negatives correctly. Since specificity is near a value of 1.0, the Youden score is almost equal to sensitivity. All three measures take into account the true negatives, which far outweigh the true positives, in our datasets, for this reason they were removed from Figure 10 and shown only in the Supporting Information. Sensitivity is much lower at 0.78 and 0.82 for experts and nonexperts respectively, as both groups partially capture the gold standard. Precision is higher for experts (0.92) than for nonexperts (0.78), meaning that experts were providing segmentations approximately the same size as the gold standard while nonexperts were providing much bigger segmentations (that generally encompass the gold standard). This explains the higher sensitivity and lower specificity of nonexperts. The Kappa and Dice score is lower for experts at 0.83 and 0.62 while the nonexperts median is 0.79 and 0.67, respectively. The Kappa score takes into account overlap with the probability of randomly obtaining the right segmentation. Given the dimensionality of our data, getting the right segmentation by accident is very low, explaining why the Kappa and Dice score are very similar. It is important to consider that the ratio of true negatives to true positives is not the same for both representations (voxels vs. streamlines).

The computation of inter‐rater reproducibility was performed using the fused triplicate to minimize the influence of intra‐rater reproducibility. The approach to fuse the triplicate is an approximation, fusing more than three segmentations from the same datasets would be necessary to perfectly evaluate intra‐rater reproducibility. However, the five datasets used for this study represent sufficiently similar tasks to consider our approximation adequate for this work. Preliminary analysis showed low correlation values, between a participant “*score*” for intra‐rater reproducibility and inter‐rater reproducibility. Correlation was between 0.2 and 0.4 for all measures, this indicates that there is no clear link between the reproducibility of a participant's own segmentations and the agreement with other participants.

## 4 DISCUSSION

### 4.1 Evaluation of protocols

This work illustrates that intra‐rater and inter‐rater agreement is far from perfect even when following a strict and “*simple*” segmentation protocol. The intra‐rater and inter‐rater agreement must be taken into account when researchers compare bundles obtained from manual segmentations. When human expertise is required for a project, it is crucial that people involved in the manual segmentation process evaluate their own reproducibility, even if they have sufficient neuroanatomy knowledge and extensive experience in manual segmentation. This measure of error could increase the threshold for statistical significance. In such case, either more datasets will be needed or a better protocol for segmentation needs to be designed (Boccardi et al., 2015; Gwet, 2012). The similarity between both groups indicates that with the right protocol, it would be possible to train people without anatomical background to perform tasks with results and quality similar to experts.

Without such evaluation, it is impossible for experts and nonexperts alike to know beforehand how reproducible they are. Since their “*scores*” vary with the protocol, the bundle of interest and possibly other factors, it is important to consider an evaluation before performing large‐scale segmentation procedure (Frisoni et al., 2015). An alternative to guarantee more reproducible results is to design an appropriate protocol for nonexperts and to perform tasks blindly more than once. The results can then be averaged, which will make outliers and errors easier to be identified. Various ideas can be considered to facilitate the tasks and increase reproducibility. Using multiple modalities, such as functional MRI or myelin map, to identify important landmarks or allowing for wider ROIs delineation by adding a safe margin around each of them could help increase reproducibility. However, requiring additional modalities would make segmentation more specific, it may be of interested to segment a bundle based only structural data (T1, dMRI). However, any modifications to the current protocol, or any protocol for that matter, would create the need for a new reproducibility assessment using a similar framework to the one presented in this work. The conclusion remains the same, the lack of quantification about the reproducibility of a protocol is by itself problematic.

This study did not allow for collaboration and did not micro‐manage participants, meaning they were left with the instructions without further intervention from the organizers. In a situation where a segmentation plan can be defined in groups and techniques can be improved along iterations of the plan, the intra‐rater and inter‐rater agreement would likely go up. This study aimed at the evaluation of participants following instructions from a protocol, similar to the ones present in books, publications or online examples.

### 4.2 Measures and representations

In this work, the intra‐rater agreement was higher for nonexperts than experts, without more information we could have concluded that nonexperts were more meticulous when they were performing their manual segmentations. However, by looking at sensitivity and precision, we can see that nonexperts had “*bigger*” segmentations. Experts are likely stricter in their decision‐making process, this could amplify the local‐decision and global‐impact conundrum mentioned earlier. A more liberal, less rigid, segmentation likely makes it easier to be reproducible but does not necessarily make it valid. This is an example showing the importance of having more than one type of measure to obtain a complete picture.

In tractography, it is common to use a single measure to portray a complex phenomenon. Most measures used are simplified to have easily interpretable results. The previous example shows the importance of using more than one type of measurements to obtain a complete picture of the reproducibility. “*Reproducibility scores*” are likely to vary with the project and the bundle of interest. This needs to be addressed as a community. The discrepancy between protocol quality, reproducibility, and conclusion put forward in the literature can be problematic.

For binary measures (accuracy and specificity), scores were both above 95% as it is easy to discard true negatives and consequently did not provide much insight. Similarly to the curse of dimensionality in machine learning (Ceotto, Tantardini, & Aspuru‐Guzik, 2011; Verleysen & François, 2005), our datasets typically contain millions of voxels (or streamlines), of which only a few thousands true positives are considered during segmentation. Thus, the vast majority of true negatives are rapidly discarded resulting in both accuracy and specificity almost reaching 100%. Sensitivity provides more information, as true positives are more difficult to get, since they are rarer in the tractograms (few thousands out of millions) (Maier‐Hein et al., 2017). This needs to be taken into account using precision, as in some cases, strict segmentation is encouraged because false positives are more problematic than false negatives. Streamline‐wise measures show lower agreement, meaning that reproducible results are likely more difficult to achieve with the streamline representation.

More complex measures need to be designed to fully capture the complexity of tractography datasets and compare them, even across datasets or for longitudinal studies. Currently, more advanced measures that capture fanning, spatial coherence, localized curvature and torsion or spectral analysis are still rare, despite being used in other neuroimaging fields (Cheng & Basser, 2018; Esmaeil‐Zadeh, Soltanian‐Zadeh, & Jafari‐Khouzani, 2010; Glozman et al., 2018; Lombaert, Grady, Polimeni, & Cheriet, 2012).

### 4.3 Tractography algorithms

Manual segmentation of deterministic tractograms is likely more reproducible, since small differences in ROI placement will have a smaller impact on the resulting bundle. The local‐decision and global‐impact conundrum mentioned earlier is more obvious with probabilistic tractography. Other tractography algorithms, such as global tractography (Christiaens et al., 2015; Kreher, Mader, & Kiselev, 2008; Mangin et al., 2013; Neher et al., 2012), are likely to have different “*reproducibility scores*,” even with the same segmentation protocol. Any change to the preprocessing could lead to unexpected change in the reproducibility “*scores*.” For the purpose of this study, we provided optimized tractograms for the bundles of interest (BOI) and our algorithmic reconstruction choices may have influenced our results. However, this is in line with our main message, which is that every project involving manual segmentation should come with its own reproducibility assessment. Hence, changing tractography algorithm would require a re‐evaluation of the reproducibility is considered as part of important future work. For example, the project “*TractEm*” (Bayrak et al., 2019) featured a framework to obtain 61 bundle of interests from deterministic tractography and report some voxel‐wise measures for intra‐rater and inter‐rater reproducibility. However, this protocol is likely optimized for specific datasets (BLSA, HCP). It also requires registration and tractograms must be generated with DSI‐Studio (deterministic) only. Any deviation from this protocol would likely change the reported reproducibility measures.

Using the same dataset and tractography algorithm, but increasing or decreasing the number of streamlines, variation in step‐size or angular threshold could also change the reproducibility “*scores*.” Another anatomical definition of the PyT having that definition taught to participants in person instead of a simple PDF document or dissections of another BOI would likely lead to different reproducibility “*scores*.” Other dataset could come with their own challenges, for example, infants or aging population, where finding anatomical landmarks could be harder and thus lead to lower reproducibility.

Such trend can be observed in numerous other studies where investigation of the same bundle, different bundles or when different algorithmic choice leads to a wide variety of reported reproducibility “*scores*” (Colon‐Perez et al., 2016; Dayan et al., 2015; Kaur et al., 2014; Kreilkamp et al., 2019; Voineskos et al., 2009; Wakana et al., 2007; Wassermann et al., 2016; Yendiki et al., 2011). Similar generalization difficulties and trends exist in the field of medical image segmentation (Boccardi et al., 2011; Frisoni et al., 2015). However, the general conclusion remains that reproducibility needs to be quantified for each specific project and protocol. Reproducibility “*scores*” cannot be easily generalized and any attempt would be dangerous, as any deviation from a known protocol creates the need for a new assessment. Aiming for standardized and harmonized protocols that are agreed upon within the field should be the main focus on the long term, such as (Bayrak et al., 2019; Catani & De Schotten, 2008).

### 4.4 Impact on analysis

If variability needs to be minimized further than the defined protocol, a simple recommendation is to have a single rater performs each task multiple times or multiple raters perform each task multiple times (or a subset of tasks). This way, it is guaranteed that each dataset is segmented more than once, decreasing the error risk. Regardless of the decision made, it is of great importance to quantify the reproducibility of manual segmentation of raters involved in the project before doing any statistics or group comparisons. This could drastically change the statistical significance of results. As of now, to the best of our knowledge, human variability and errors are rarely considered. Measurements such as volume and streamlines count can take into account the measurement error (i.e., voxel‐wise or streamline‐wise Dice coefficient) as part of group variances. Combining the measurement errors with a group average can be achieved by using, for example, the principle of pooled variance (Gwet, 2012; Peters, 2001). However, if the intra‐rater “*reproducibility score*” is too low, for example, below 30%, reporting such measurements is counterproductive, as this will require much larger cohorts to reach statistical significance. Taking into account the measurement error makes sense as long as the same “*thing*” is being measured.

Sources of variability need to be accounted for to truly enable synthesis of work across multiple centers. Even when automatic or semi‐automatic methods are used, they first need to be evaluated with agreed upon measures and reach or surpass human standards. In a very thorough longitudinal large‐scale project across centers involving manual segmentation, it would be desirable to acknowledge the variability across timepoints, across scanners, and across rater into the analysis.

The extension to other bundles of interest or other segmentation plans is not trivial and the only conclusion that stands is that agreement is never 100% and that a unique measure is not enough to represent the whole picture for tractography segmentation. The desire to simplify measures or have only one value to describe quality or reproducibility of segmentations needs to be discouraged. The nature of our datasets makes this task much more complex to interpret than 2D or 3D images, and it is imperative that the field comes to understand and agree on measures to report. This is more relevant than ever as the field grows and now that open science is becoming more popular and reproducibility studies are encouraged. Similarly to other neuroimaging fields, such as hippocampi segmentation, standardized protocols need to be developed and designed to be used across multiple centers without active collaboration or micromanagement.

### 4.5 Future work

Future work includes the creation of a database containing bundle segmentations and metadata from participants that will be available online so further analysis can be done. This metadata could help explain the variability, similar trend has been observed for most types of measurements, medical images, or tractography segmentation (Boccardi et al., 2011; Bürgel et al., 2009; Gwet, 2012). As for now, a preliminary upload of the participants segmentation is available on Zenodo (https://doi.org/10.5281/zenodo.2547024), which will be updated. In this work, metadata was not used to evaluate duration as a variable influencing reproducibility. Investigating the relationship between variability and duration of a task or looking for bias (inter‐hemispheric or software influence) could be of interest for future research. An online platform similar to the Tractometer (Côté et al., 2013) or a Nextflow pipeline (Di Tommaso et al., 2017) is planned to be released. Such a tool would be designed for researchers to quickly submit data that is expected to have some level of agreement and obtain their “*reproducibility score*.” This way protocols can be improved and reproducibility can be taken into account in the analysis.

Protocols for many bundles need to be developed for various purposes, such as clinical practice, synthesis of findings, and building training sets for machine learning. The segmentation plan and instructions need to be defined clearly by panels of experts, and agreed upon terminology (Mandonnet, Sarubbo, & Petit, 2018), to optimize reproducibility and anatomical validity. The field of manual tractography segmentation is decades behind fields such as gray nuclei or hippocampi manual segmentation on this matter. The latter has been refining segmentation protocols for the past decade and has already reached the state harmonized segmentation protocol and was evaluated with reproducibility in various settings (Apostolova et al., 2015; Boccardi et al., 2011, 2015; Frisoni et al., 2015; Wisse et al., 2017).

## 5 CONCLUSIONS

When trying to understand how similar WM bundles from dMRI tractography are, at least three values need to be taken into consideration: *Dice coefficient of voxels* showing how well the overall volume overlaps, *Dice coefficient of streamlines* showing if the same streamlines were selected/discarded, and *correlation of density map* showing if the streamlines are spatially distributed in a similar way. Results specific to our work on the pyramidal tract revealed that rater overlap is higher for voxel‐wise measures (approximately 70%) than streamline‐wise measures (approximately 20%).

In comparison to the group average, the results depict an ease to identify true negatives, an adequate number of true positives, while having a low number of false positives. The voxel and streamline representations do not produce equal levels of reproducibility. Studies reporting bundle asymmetry in terms of streamline count (streamline based) will require a larger group difference than those reporting volume difference (voxel based). Our particular protocol served as a powerful illustration of the importance of assessing the variability of human expertise when comparing population and provides interesting insights on WM manual segmentation.

The lack of framework for reproducibility assessment, the sparse literature on intra‐rater and inter‐rater variability in tractography and the variation in the reported values across bundles, reconstructions, datasets and other variables points to the importance our proposed framework for evaluation, as a step forward. It is of importance to reiterate that the intention of this study is not to propose/enforce a processing pipeline for tractography and/or propose a new set of rules for PyT segmentation. The diversity of reported values for reproducibility shows that clearly defined processing and segmentation protocol is necessary in this field. All data and metadata used in this work are now publicly available (https://doi.org/10.5281/zenodo.2547024) in the hope to stimulate discussions and more evaluations in the future for other bundles and protocols. Better reproducibility of results is needed and goes hand‐in‐hand with the open science movement. A collaborative effort to evaluate and quantify human variability is needed.

## Data Availability

The data that support the findings of this study are openly available in on Zenodo at https://doi.org/10.5281/zenodo.2547024
